# Calibration of Kinect for Xbox One and Comparison between the Two Generations of Microsoft Sensors

**DOI:** 10.3390/s151127569

**Published:** 2015-10-30

**Authors:** Diana Pagliari, Livio Pinto

**Affiliations:** Politecnico di Milano, Department of Civil and Environmental Engineering (DICA)-Geomatic and Geodesy Section, Piazza Leonardo da Vinci 32, 20133 Milan, Italy; E-Mail: livio.pinto@polimi.it

**Keywords:** Kinect, calibration, depth maps, distortion removal, RGB-D, fusion libraries

## Abstract

In recent years, the videogame industry has been characterized by a great boost in gesture recognition and motion tracking, following the increasing request of creating immersive game experiences. The Microsoft Kinect sensor allows acquiring RGB, IR and depth images with a high frame rate. Because of the complementary nature of the information provided, it has proved an attractive resource for researchers with very different backgrounds. In summer 2014, Microsoft launched a new generation of Kinect on the market, based on time-of-flight technology. This paper proposes a calibration of Kinect for Xbox One imaging sensors, focusing on the depth camera. The mathematical model that describes the error committed by the sensor as a function of the distance between the sensor itself and the object has been estimated. All the analyses presented here have been conducted for both generations of Kinect, in order to quantify the improvements that characterize every single imaging sensor. Experimental results show that the quality of the delivered model improved applying the proposed calibration procedure, which is applicable to both point clouds and the mesh model created with the Microsoft Fusion Libraries.

## 1. Introduction

3D scene modeling, gesture recognition and motion tracking are active and quickly developing research sectors. The videogame industry has been driven by a recent boost in the field of gesture recognition, to make the game experience for users more immersive and fun. Starting from the idea of creating a sensor that allows users to play without holding any controller, Microsoft Corporation developed the Kinect sensor. The Kinect was first launched on 4 November 2010 as an accessory for the Xbox360 console. It was developed by Microsoft and the Israeli company PrimeSense and it was entered the Guinness World Records as the fastest selling consumer device, with 8 million units sold during the first 60 days on the market.

The Kinect allows new interactions during games, based on the use of gesture and voice. Since its presentation it has attracted researchers from different fields, from robotics [1,2,3,4,5] to biomedical engineering [6,7,8,9] and computer vision [10,11,12,13]. Shortly after the Kinect launch, the device was hacked and Software Development Kits (SDKs) created by third party communities have spread throughout the Web, permitting the sensor to be used not only as a game device, but also as a measurement system. See for example [14,15,16,17]. On 16 June 2011, the official Microsoft SDK was released.

This gaming control device has had large success in various fields because it extends the technology of depth cameras to low-budget projects. In fact, the complementary nature of the information provided by the Kinect (depth and visual images) has established new solutions to solve old problems with new approaches [11] by combining geometry and visual information.

The original Kinect consisted of an RGB camera, an IR emitter and an IR camera. It is capable of acquiring color and depth images of the scene. Depth measurements are performed using speckle pattern technology [18]. On March 2013, the Kinect Fusion libraries were released. They allow reconstructing in real time 3D scene by simply holding in hand the Kinect and moving it in space. The system integrates consecutive depth data, assuming that the relative position between the sensor and the object can be continuously tracked, reconstructing a single 3D model. More details on how the Fusion Libraries work can be found in [19]. In the summer of 2014, a new generation of Kinect was introduced to the market. This new sensor is more precise and it is based on time-of-flight technology. 

As noted before, the Kinect is a low-cost sensor, originally created to be a gaming control device. For this reason it is fundamental to investigate its accuracy and precision, in order to define the expected quality of the measurement as a function of the distance between the frame object and the sensor itself. Moreover, it is important to define possible systematic errors that can be corrected by a calibration procedure. A lot of work has been done for the first generation of Kinect [14,15,20,21,22,23], but to our knowledge very little information is available for the second generation of sensors [24,25]. Nevertheless, this sensor has been advertised to offer great advantages over Kinect for Xbox360, so it is important to quantify this improvement in resolution.

## 2. The Two Generation of Kinect Sensors

The Kinect sensor for Xbox360 (from now on Kinect 1.0) is an active camera. Unlike other human-based control devices lunched by other firms (see for examples, Wii Remote Control by Nintendo or PlayStation Move by Sony) it allows users to play and completely control the console without having to hold any kind of device, but only by the use of voice and gesture. The Kinect is a low-cost sensor that allows the real-time measurement of depth information (by triangulation) and the acquisition of RGB and IR images at a frame rate up to 30 fps. It is composed of an RGB camera, an IR camera, an IR-based projector, a microphone array, a tilt motor and a 3-axis accelerometer. 

Kinect 1.0 measures distances using a coded light technique [26]. The IR projector emits a speckle pattern on the frame scene; the IR camera captures the reflected pattern and computes the corresponding depth for each image pixel. The depth measurement is performed by a triangulation process, as described in [14]. The observed quantity is the disparity, which corresponds to the shift necessary to match the pattern captured by the IR camera with the reference model.

**Figure 1:**
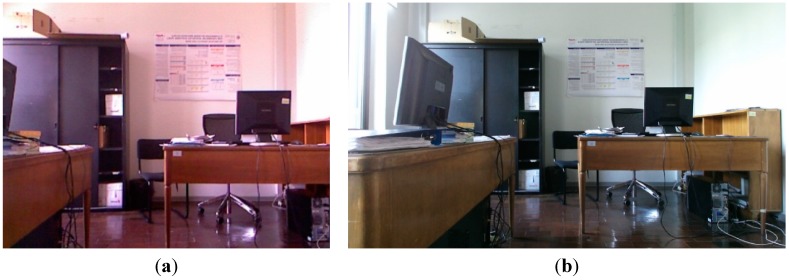
Kinect 1.0 RGB image (**a**) compared with Kinect 2.0 RGB image; (**b**) The two images have been acquired from the same standpoint.

**Figure 2:**
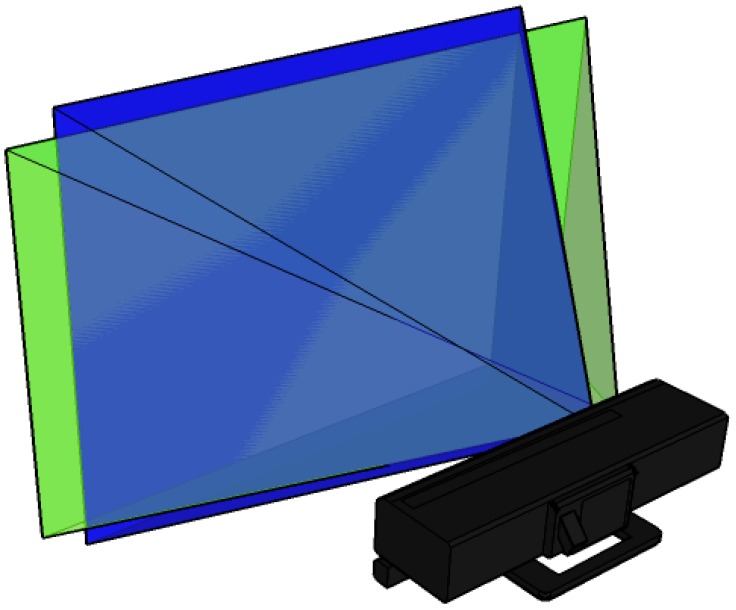
Kinect 2.0 RGB and IR cameras FOV. In blue is represented the FOVs of the IR camera, while in green is represented the FOVs of the RGB camera.

The main drawback of the Kinect 1.0 sensor is the low geometric quality of the delivered data, noise and low repeatability. The RGB has poor quality, comparable to that of webcams. The depth data registered by the Kinect 1.0 has poor quality too, due to the fact that the structured light approach is not always robust enough to provide a high level of completeness of the framed scene. In fact, information extracted from a single acquisition is usually stepped and it is delivered with missing parts. Moreover, the data registered by the sensor is very noisy, as it is better explain in following Section 3.2. To provide high resolution image and depth data, a second generation of Kinect has been released. It provides better depth measurements, in order to perform more precise skeleton tracking and gesture recognition. Kinect 2.0 has the same number of sensors as the Kinect 1.0; however, depth is measured with a completely different measurement principle. RGB images are acquired in High Definition (HD). For a direct comparison between the RGB images delivered by the two generation of Kinect devices, see Figure 1. RGB and IR images acquired with the Kinect 2.0 partially overlap, since the new color camera has a wider horizontal Field of View (FOV), while the new IR camera has a larger vertical FOV (see Figure 2). Kinect 2.0 is defined by Microsoft as a time-of-flight system. Actually, the observed quantity is a phase measurement, so it is not completely correct to define it as a time-of-flight sensor. However, we decided to maintain this terminology to be consistent with the available literature that describes the sensor. The main characteristic of the two versions of the Kinect are illustrate in Table 1.

**Table 1:** Comparison between Kinect 1.0 and Kinect 2.0 main characteristics.

	Kinect 1.0	Kinect 2.0
RGB camera (pixel)	1280 × 1024 or 640 × 480	1920 × 1080
Depth camera (pixel)	640 × 480	512 × 424
Max depth distance (m)	4.0	4.5
Min depth distance (m)	0.8	0.5
Horizontal FOV (degrees)	57	70
Vertical FOV (degrees)	43	60
Tilt motor	Yes	No
Skeleton joint define	20	26
Full skeleton tracking	2	6
USB	2.0	3.0
Price (€)	80	199

The operating principle behind the time-of-flight sensor is described in [27]. The main feature is that the sensor is made by differential pixels, meaning that each pixel is split in two accumulators and a clock regulates which one of the pixel side is the one currently active. This permits creating a series of different output images (depth images, grey scale images dependent from ambient lighting and grey scale images independent from ambient lighting).

The system measures the phase shift of the modulated signal and computes depth from phase using Equation (1):

(1) $$2d=\frac{phase}{2\pi }\cdot \frac{c}{f_{mod}}$$

where d is the depth measure, c is the speed of light, $f_{mod}$ is the modulation frequency.

Kinect 2.0 acquires images at multiple frequencies, eliminating the ambiguity of depth measurements. The frequencies used by the sensor are approximately 120 MHz, 80 MHz and 16 MHz. The time-of-flight Kinect 2.0 sensor relies on measuring the differences between two accumulators, each one containing a portion of the returning IR light. If the scene has low ambient IR light, the sensor works properly outdoors. However, under direct sunlight radiation it could be difficult to differentiate the emitted IR pulse from the background signal, because the difference of the radiation captured by the two accumulators is small, when compared to the overall amount of incoming IR light.

Furthermore, under direct sunlight, the quality of the data delivered by Kinect 2.0 strongly depends on the incidence angle of the sunlight [25]. Nevertheless, some studies in the literature evaluating the feasibility of the use of this sensor in outdoor conditions have been presented.

The experiments in [28] tested both the Kinect versions outdoors underlying how the pattern projected by Kinect 1.0 is strongly altered by sunlight and how the new sensor is less sensitive (if not entirely immune) to this kind of radiation. Kinect 2.0 delivers point clouds even in situations where the previous version was unable to produce any results, like sunlit walls or cars. Reference [29] underlines the great potential of Kinect 2.0 for low-cost costal mapping. This work, also discussed the use of the sensor for underwater applications, verifying that for depths greater than one meter the delivered data becomes very fuzzy and incomplete and then faded away entirely. 

## 3. Geometric Calibration of the Optical Sensors

As stated before, the Kinect is a low cost sensor and its main drawback (especially for version 1.0) is the poor geometric quality of the 3D data and the low repeatability to produce accurate results. For instance, if one compares different subsequent depth frames acquired without moving the sensor it is common to have different measurements for the same pixel or even no-data. 

In order to evaluate the accuracy and the repeatability of the data, as well as test the improvement of the new version of the sensor when compared to the original version, a series of experiments have been carried out.

### 3.1. RGB and IR Camera Calibration

The first series of tests have been conducted to determine the calibration parameters of both Kinect versions. A standard camera calibration was performed to determine the Interior Orientation (IO) parameters (focal length, position of the principal point and the coefficients that describes the lens distortion) of both RGB and IR camera using PhotoModeler^®^ software, version 2012.2.1.780 [30]. In order to avoid interference between the projected speckle pattern and the camera calibration target recognition tool, the IR projector of Kinect 1.0 has been covered. A specific Graphic User Interface (GUI) was coded to control the sensor and show the video stream on the screen of the computer. The software can be used to grab single video frames, so it is possible to rotate and translate the sensor in the corrected position and acquire only the desired frames. 

In order to compute the camera intrinsics, the PhotoModeler^®^ Camera Calibration tool requires some initial guess to be used to scale the problem (because the size of the calibration polygon is unknown); usually this data are extracted from the EXIF file, but the images delivered by the Kinect lack this information. This means that the dimensions of the camera sensor have to be collected from a different source. The data from Kinect 1.0 can be easily found (see for example [15]), but Kinect 2.0 is relatively new on the market and Microsoft has not yet released all the information about the imaging sensors. Some data about the depth camera is available in [31], but to our knowledge, no information has been released about the dimension of the RGB camera sensor. For this reason the parameters estimated for this camera are corrected, up to a scale factor. The IO parameters estimated during the calibration procedure are reported in Table 2 and Table 3, respectively for Kinects 1.0 and 2.0. 

**Table 2:** Sensors and IO parameters of RGB and IR cameras estimated during the camera calibration procedure for Kinect 1.0.

Camera Name				
	Kinect 1.0 RGB Camera		Kinect 1.0 IR Camera	
**Imaging Sensor**				
Type	Aptina MT9M112 CMOS		Aptina MT9M001 CMOS	
Resolution (pixels)	1280 × 1024 or 640 × 480		640 × 480	
Pixel size (µm)	2.8		5.2	
**Interior Parameters**				
	Value	St. Dev	Value	St. Dev
Focal length (mm)	3.099	2.0e−3	6.497	3.0e−3
Format width (mm)	3.58		6.66	
Format height (mm)	2.87		5.32	
Image width (pixels)	640		640	
Image height (pixels)	480		480	
Principal Point *x* (mm)	−0.040	9.2e−4	−0.005	2.0e−3
Principal Point *y* (mm)	−0.020	1.0e−3	−0.004	3.0e−3
**Additional Parameters**				
K_1_ (mm^−2^)	−1.366e−3	9.1e−5	1.795e−3	4.3e−5
K_2_ (mm^−4^)	7.857e−4	1.7e−5	−8.337e−5	2.5e−6
P_1_ (mm^−2^)	−1.518e−4	2.9e−5	−1.835e−4	2.1e−5
P_2_ (mm^−2^)	−9.514e−4	3.2e−5	2.538e−4	2.2e−5

**Table 3:** Sensors and IO parameters of RGB and IR cameras estimated during the camera calibration procedure for Kinect 2.0.

Camera Name				
	Kinect 2.0 RGB Camera		Kinect 2.0 IR Camera	
**Imaging Sensor**				
Type	-		-	
Resolution (pixels)	1920 × 1080		512 × 424	
Pixel size (µm)	3.1		10	
**Interior Parameters**				
	Value	St. Dev	Value	St. Dev
Focal length (mm)	3.291	1.0e−3	3.657	5.2e−4
Format width (mm)	6.00		5.12	
Format height (mm)	3.38		4.24	
Image width (pixels)	1920		512	
Image height (pixels)	1080		424	
Principal Point *x* (mm)	−0.005	5.6e−4	0.032	3.5e−4
Principal Point *y* (mm)	−0.016	6.9e−04	0.033	3.9e−4
**Additional Parameters**				
K_1_ (mm^−2^)	3.823e−3	3.8e−5	−6.510e−3	2.7e−5
K_2_ (mm^−4^)	3.149−4	3.8e−6	1.205e−3	3.8e−6
P_1_ (mm^−2^)	2.332e−4	2.0e−5	1.377e−4	8.0e−6
P_2_ (mm^−2^)	−5.152e−4	2.1e−6	1.589e−4	9.2e−6

Other authors have already faced the problem of calibrating the Kinect 2.0. The results reported in Table 3 are comparable with those reported in [24,29]. However, the results discussed in this paper have been estimated with better precisions and all the distortion parameters are significant.

### 3.2. Image Sensors Precision 

In order to evaluate the precision of the Kinect the different data delivered by the two versions of the sensor (RGB, IR images and depth measurements) were statistically analyzed. To this end, 100 subsequent frames were captured with each single camera. The image frame rate used was equal to 30 fps, for Kinect 1.0, and to 15 fps for Kinect 2.0 (these were the maximum possible frame rates achievable with the hardware used: HP 15-j100el laptop with Intel Core i7-4700MQ processor, NVIDIA GeForce GT 740M graphic card with 2 GB-DDR3 of dedicated memory and 750 GB (5400 rpm) SATA Hard Drive). Using such high acquisition rates, it is possible to assume that no environmental changes (*i.e*., illumination or temperature variations) occurred during the test period. Likewise, it is reasonable to assume that there were not variations related to the internal temperature of the sensor: as shown by [24,32], after a first pre-heating of the Kinect, the influence of temperature on the measured distances is a long-term effect, mainly linked to the switching on and off of the cooling fan.

**Figure 3:**
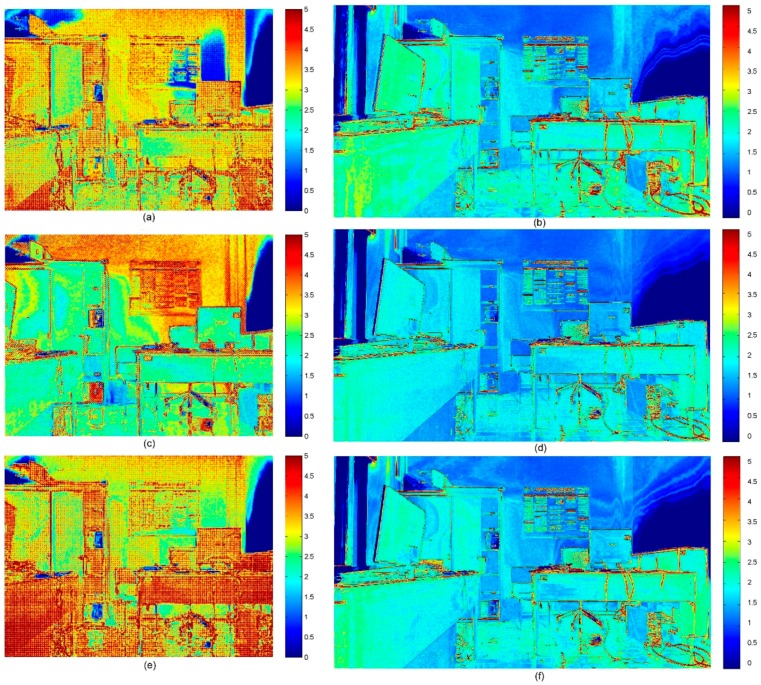
Standard deviation (st.dev.) computed on 100 subsequent frames acquired with Kinect 1.0 (left) and Kinect 2.0 (right) for each corresponding pixel for each channel. (**a**) Kinect 1.0 st.dev.: red channel; (**b**) Kinect 2.0 st.dev.: red channel; (**c**) Kinect 1.0 st.dev.: green channel; (**d**) Kinect 2.0 st.dev.: green channel; (**e**) Kinect 1.0 st.dev.: blue channel; and (**f**) Kinect 2.0 st.dev.: blue channel.

Figure 3 shows the color maps representing the standard deviation in an 8-bit color depth scale (256 tonal values) for both Kinect sensors. On the left, the color maps obtained for Kinect 1.0 are reported, while on the right are reported those obtained for Kinect 2.0. It is quite clear that there is a certain level of variation of the registered intensity value, especially in correspondence of object boundaries, for both sensor versions. However, it is evident that the images acquired with the new generation of sensors are much more stable in each one of the single RGB channels. It is also interesting to notice how the green channel is characterized by lower variations, probably because the elements sensitive to green light are, in the Bayer pattern, double of those sensible to blue or red light. The sensor stability analysis was performed also for IR camera. It is worth noting that the larger standard deviations are probably due to the data stored using 16 bit (65,536 tonal values).

**Figure 4:**
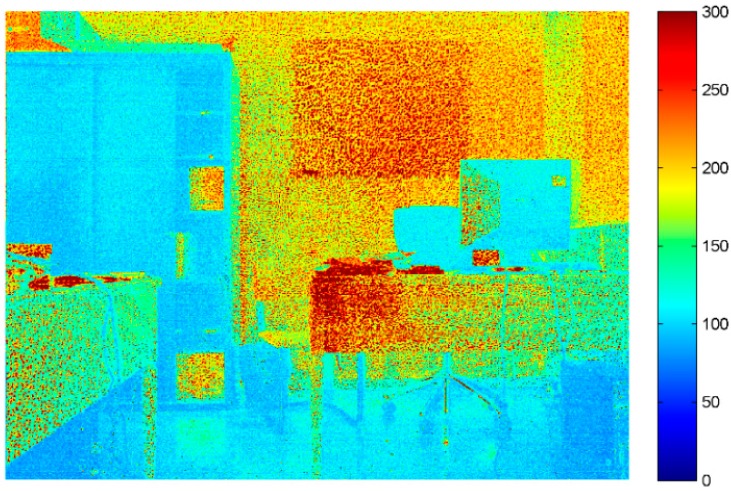
Standard deviation computed for each corresponding pixel for 100 subsequent IR images for Kinect 1.0.

**Figure 5:**
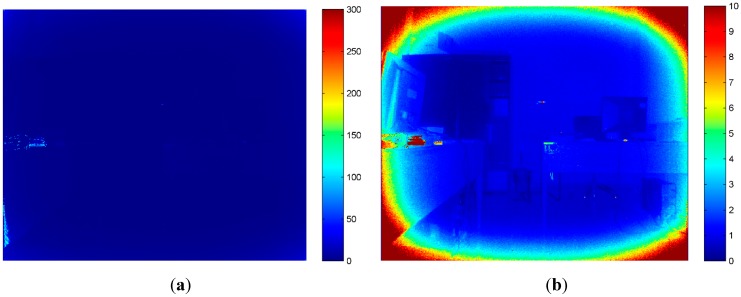
Standard deviation computed for each corresponding pixel for 100 subsequent IR images for Kinect 2.0. (**a**) It is the map obtained using the same scale used for Kinect 1.0; (**b**) The same image has been saturated using a different color scale, revealing an interesting radial phenomena.

In Figure 4 and Figure 5 the color maps computed for the IR images acquired for the two version of the Kinect are represented. The color map computed from the images acquired with the second-generation sensor is clearly more stable and less fuzzy, mainly because the time-of-flight technology does not require projecting any random pattern (which is clearly visible in Figure 4). Figure 5 illustrates, on the right, the same map that is presented on the left side of the figure, but with a more appropriate color scale range. A radial effect is clearly visible. However, these variations are in the order of 10 tonal values in a 16-bit representation, so the IR images delivered by Kinect 2.0 can be considered very stable, demonstrating how the new measurement technique represents a huge improvement over the structured light ones.

**Figure 6:**
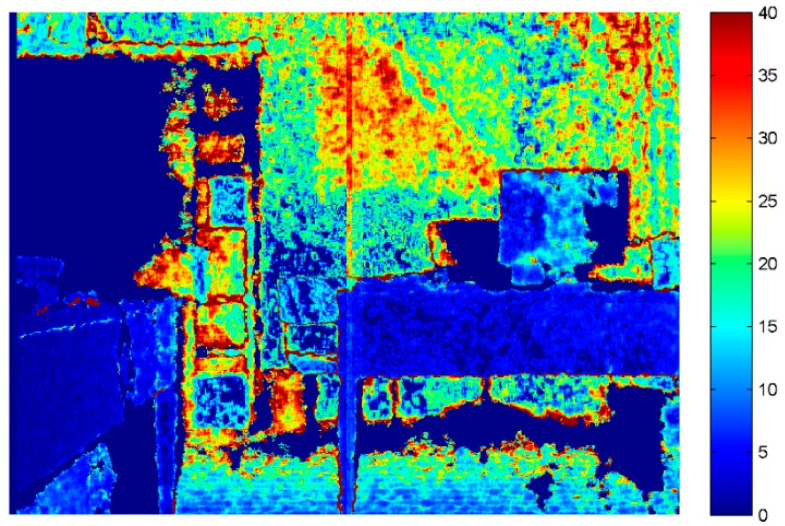
Standard deviation (mm) computed for each corresponding pixel of the raw depth data acquired by Kinect 1.0 IR camera.

**Figure 7:**
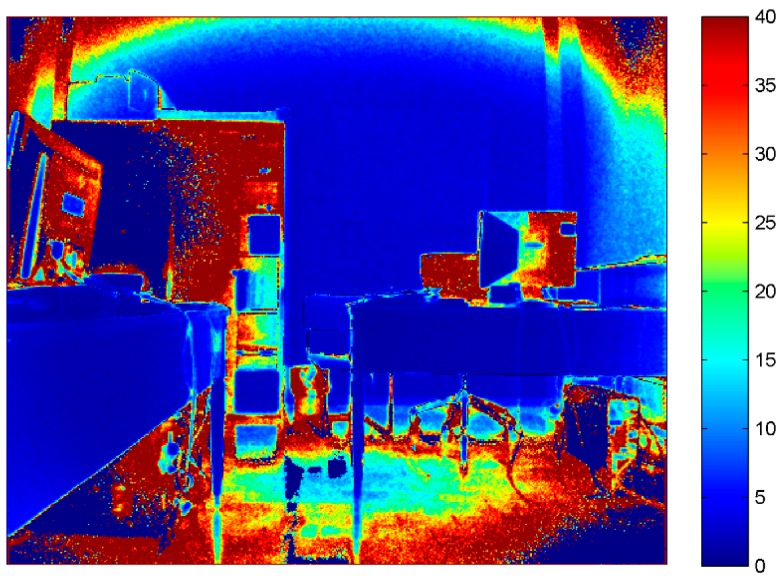
Standard deviation (mm) computed for each corresponding pixel of the raw depth data acquired by Kinect 2.0 IR camera.

As previously done for RGB and IR images, the mean and the standard deviation of each corresponding pixel on different frames were computed for depth images too. The sensor delivers data equal to zero when it is not able to perform any measurement at all, therefore null values have been removed from the computations. In Figure 6 and Figure 7, the standard deviation maps created from depth measurement delivered by the two Kinect versions are shown. Comparing the colored maps reported in Figure 6 and Figure 7, it is quite evident that the new version of the device is more precise around the object border, but the quality of the depth measurement is predominantly a function of the object reflective properties.

**Figure 8:**
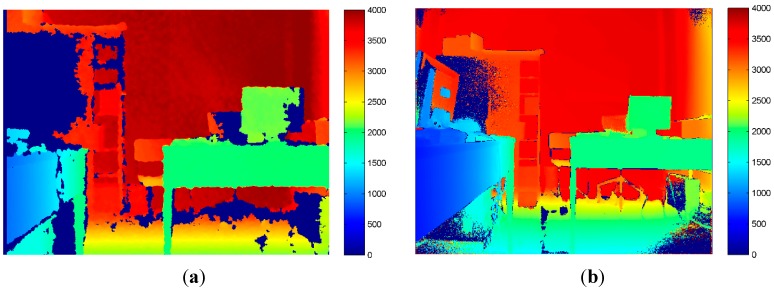
Depth maps delivered by Kinect 1.0 (**a**) and Kinect 2.0; (**b**) (mm). In dark blue are represented the no-data value delivered by the sensors.

However, it is worth noting the remarkable improvement of the new generation of Kinect in the reduction of the no-data values delivered while performing depth measurement, represented by dark blue in Figure 8. Moreover, the depth maps delivered by Kinect 2.0 provide a higher number of visible details. Nevertheless, for Kinect 2.0 the presence of a radial effect is quite evident.

### 3.3. Depth Image Distortion Correction 

Kinect 2.0 IR and depth images are measured by the same imaging sensor, by performing different computations on the response of the differential pixel of the CMOS sensor [27]. This means that the two images are co-registered and the optics are the same. Starting from this consideration the distortion of each depth image has been removed applying the Brown model [33], using the coefficient estimated for the IR camera during the calibration procedure (reported in Table 3). The validity of this procedure has been tested by evaluating the standard deviation of the individual pixels in a 100 frames sequence acquired without moving the Kinect. Objects of different shape and sizes, located at different distances were placed in the imaged scene. The average of the standard deviations computed after the distortion removal decreases, mainly because during the image resampling the scene is better reconstructed, especially with respect to high depth gradient correspondence. 

### 3.4. Depth Measurement Accuracy 

The Kinect 1.0 depth camera is able to collect data in the range 0.80–4.00 m, but because the baseline between the IR camera and the IR pattern projector is very short (around 0.074 m) it is important to quantify the error committed by the sensor when the distance from the object increases. The Kinect 2.0 depth camera can acquire data in the range 0.50–4.50 m. It is based on a different measurement principle, and being a relatively new sensor on the market, it is important to evaluate its potential and weaknesses too. 

A straightforward calibration procedure was performed to estimate the error of the two versions of the sensor, as a function of the distance from the object. During the acquisitions, the sensors were located at known distances from the wall chosen as a reference plane. Reference distances were measured with a laser distance meter placed at the two extremities of the sensor, in order to limit some possible rotation effects. For each position, 100 depth images have been acquired. The data were stored as 16-bit images, to get a discretization equal to the sensor resolution (0.001 m). The sensors were progressively moved away from the wall, from 0.80 to 4.00 m, with regular steps (on average 0.40 m). 

In order to quickly correct the data directly within the Microsoft libraries, a fast and simple procedure has been developed, using a single function that describes the sensor error as a function of the distance from the objects. In order to verify the parallelism between the sensor plane and the wall, the differences between the interpolating plane and a *z*-constant plane (equal to the average measured distance) have been computed. In all the cases the differences were lower than the sensor precision, therefore it was not necessary to roto-translate the depth maps to correct the residual rotation error.

As the Kinect was moved further away, other elements such as the floor appeared in the images. Therefore, the statistical analysis was conducted by selecting a window of 200 × 200 pixels, located as centered as possible on the image frame in order to discard border effects, and corresponding only to the wall chosen as the reference plane. For each acquisition step the average and the standard deviation of each corresponding pixel (for the selected patch on the 100 images) were computed.

Using this procedure a correction function has been estimated. It can be applied to the whole image, given a specific distance. Some tests have been conducted to evaluate the effects of intrinsics, extrinsics and depth frames correction on the 3D model created using the Microsoft Fusion Libraries. Depth correction has emerged to be the most influent and efficient one to quickly correct systematic errors that characterize the Kinect 1.0 [34].

**Figure 9:**
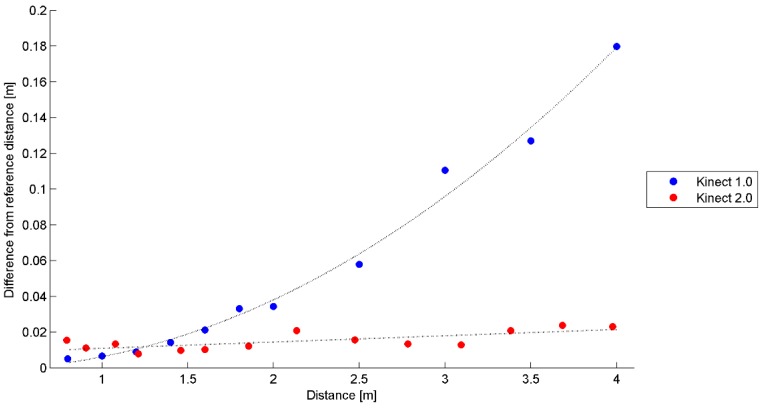
Estimation of the error committed by the two Kinect sensors as a function of the distance between the device and the object.

Figure 9 shows the estimation of the depth error of the two versions of the Kinect sensors as a function of the distance between the sensor and the object. It is evident that the distance measurements delivered by the new Kinect sensor are much more precise than the ones performed by the previous one. For instance, at a distance equal to 3 m, the Kinect 2.0 error is equal to 0.02 m, while the Kinect 1.0 error is in the order of 0.1 m.

The interpolating function used for Kinect 1.0 is a second order polynomial function. The analytical expression that gives the distance error of this sensor can be deduced from the general case that describes the error along the direction orthogonal to the sensors, considering the relative orientation between two cameras. Considering a pseudo-nadiral geometric configuration, the depth error (in this case the measured distance) can be described by Equation (2) [35]:

(2) $$dZ=-\frac{Z}{b}db_{x}-\frac{X-b}{b}db_{z}-\left(\frac{Z^{2}}{b}+\frac{X^{2}}{b}\right)d\varphi_{1}+\frac{XY}{b}d\omega_{1}-\frac{YZ}{b}d\kappa_{1}+\left(\frac{Z^{2}}{b}+\frac{{\left(X-b\right)}^{2}}{b}\right)d\varphi_{2}+\frac{\left(X-b\right)Y}{b}d\omega_{2}-\frac{YZ}{b}d\kappa_{2}$$

where b is the baseline between the IR projector and IR camera center, X, Y and Z are the object coordinates, $db_{x}$, $db_{z}$, $d\varphi_{1}$, $d\omega_{1}$, $d\kappa_{1}$, $d\varphi_{2}$, $d\omega_{2}$ and $d\kappa_{2}$ are the relative orientation parameters errors, with ω, φ, κ representing the gimbal angles. It can be easily seen that an error in φ, corresponding to a residual rotation along the vertical axis between the two cameras, introduces an erroneous estimation of depth that varies quadratically with Z coordinate (that represents the measured depth). 

On the contrary, the interpolating function used for Kinect 2.0 sensor is a linear one. Starting from the general expression that describes the measurement principle (see Equation (3)) of a distance meter based on phase-shift principle the depth error committed by Kinect 2.0 can be described as [36]:

(3) $$Z=\frac{n}{2}\frac{c}{f_{mod}}+\frac{\phi }{4\pi }\cdot \frac{c}{f_{mod}}$$

where c represents the speed of light in air, n is the integer number of phase cycle, Z the measured distance, $f_{mod}$ the modulation frequency of the emitted signal and ϕ the measured phase of the returning signal. 

The error committed by Kinect 2.0 can be described as:

(4) $$dZ=\frac{-Z}{f_{mod}}df_{mod}+\frac{Z}{2n\pi +\phi }d\phi$$

Equation (4) describes how an erroneous phase measurement, as well as an error during the modulation of the emitted signal, varies linearly with the measured depth Z.

It is important to notice how Equations (3) and (4) do not consider the residual error between the two distance measurement systems, because the origin of the Kinect one is not known. This can be considered a second order effect which does not affect the shape of the estimated functions, but only translates them downward by a quantity equal to the instrumental zero point.

The interpolated functions used to describe the error committed by the two version of the Kinect have been estimated via Least Squares Methods and the significance of the estimated parameters has been verified by performing a Student’s *t*-test. A significance level equal to 5% for all the estimated parameters verified that the functions correctly fit the experimental data.

### 3.5. Depth Measurement Precision 

In order to define the sensor noise as a function of the distance between the sensor and the object, the average standard deviation for each acquisition step was computed, considering a 200 × 200 pixel window covering a flat area orthogonal to sensor axis. 

**Figure 10:**
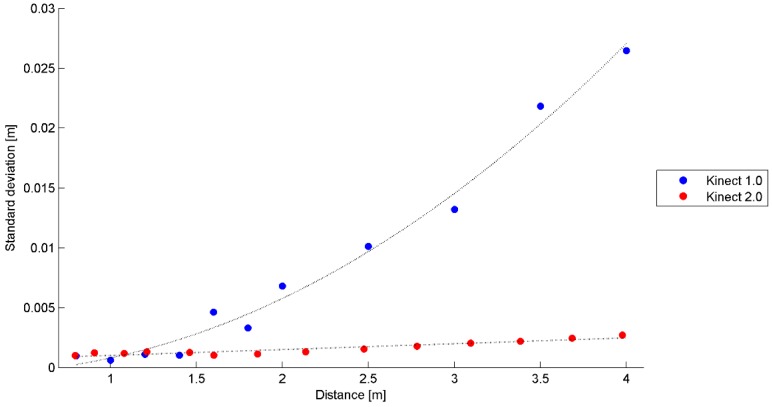
Standard deviation of the depth measurement performed by the two Kinect sensors as a function of the measured distance.

In Figure 10 the average of the standard deviations of the 200 × 200 patch computed for the two Kinect versions, as a function of the distance between the system and the object, is shown.

From the experimental results reported in Figure 10, it is evident that the noise of Kinect 2.0 is quite small and that at distance of 3.5 m it is one order of magnitude lower than that of the Kinect 1.0.

The interpolating function used to characterizing the noise of the Kinect 1.0 is a second order polynomial function, as one would expect from a triangulation system. In fact, a point located at distance Z from the device is characterized by a precision $\sigma_{Z}$, described by well-known Equation (5):

(5) $$\sigma_{Z}=\frac{Z^{2}}{f\cdot b}\sigma_{d}$$

where Z is the measured distance, f is the focal length of the IR imaging device, b is the baseline between the perspective center of the projector and the imaging device and $\sigma_{d}$ is the precision of observed disparity d.

The sensor noise of Kinect 2.0 can be described with a linear function. Starting from Equation (3), it is possible to derive the precision of the depth measurement ${\sigma_{Z}}^{2}$:

(6) $${\sigma_{Z}}^{2}={\left(\frac{-Z}{f_{mod}}\right)}^{2}{\sigma_{f_{mod}}}^{2}+{\left(\frac{Z}{2n\pi +\phi }\right)}^{2}{\sigma_{\phi }}^{2}$$

where c represents the speed of light in air, n is the integer number of phase cycles, Z the measured distance, $f_{mod}$ the modulation frequency of the emitted signal, ϕ is the measured phase of the returning signal, $\sigma_{f_{mod}}$ is the precision of the modulation frequency and $\sigma_{\phi }$ is the precision of the measured phase. Considering the first term of Equation 6, it is evident how the sensor noise is linearly dependent on the measured distance, in agreement with the experimental results obtained during sensor calibration and presented in Figure 10. 

Also in this case the significance of the estimated parameters of the interpolation polynomial function has been tested with a Student’s *t*-test. All the estimated parameters have been found to be significant.

From the analysis presented so far, the second generation of Kinect is more accurate and precise than the sensors of the first one. For this reason further analysis has been conducted for the Kinect 2.0, to analyze also second order effects.

**Figure 11:**
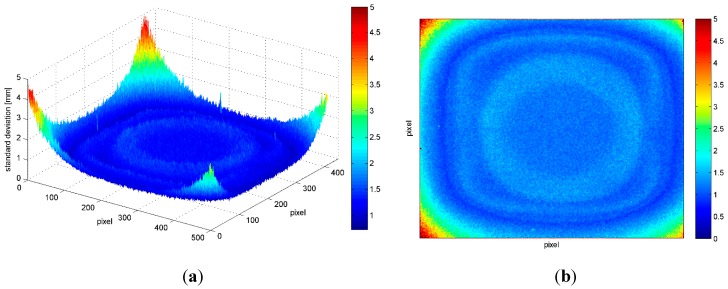
Standard deviations (mm) computed at each pixel from 100 acquisitions of a flat surface, acquired at a distance of 0.9 m. The sensor image plane was parallel to the object surface.

In Figure 11, the standard deviations computed for each pixel considering 100 acquisitions of a flat surface from a distance of 0.9 m are shown. The standard deviation increases sharply at the corners (up to 0.005 m) while in the rest of the image the variation is smaller (it oscillates between 0.001 and 0.002 m with a roughly radial symmetry). This effect is present also in the undistorted images, such as the ones reported in Figure 11. However, it is important to notice that the standard deviation variations are lower than the tolerance of the Kinect (corresponding to 0.002 m and represented in blue) for the large majority of the sensor area, therefor it can be neglected for the large majority of applications.

**Figure 12:**
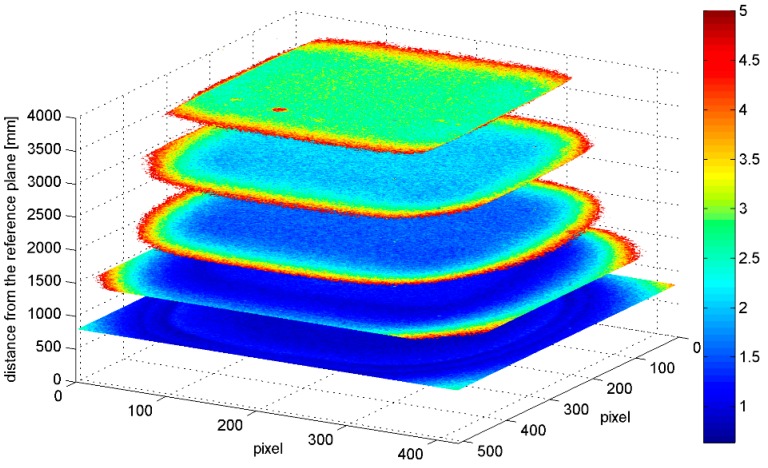
Standard deviation (mm) of the corresponding pixels computed considering images acquired at different distances from a flat surface. The *x* and *y*-axis represent the image frame, while along the *z*-axis the distances from the reference plane are reported. The color scale represents the standard deviation value.

Figure 12 gives a 3-dimensional representation of the standard deviation of the corresponding pixels computed considering images acquired at different distances from a flat surface. Only pixels with standard deviation value equal or lower than 0.005 m have been considered; this threshold value has been selected considering it as the limit for medium quality 3D modelling. From this representation two interesting considerations arise. As noted in Figure 10, the standard deviation increases for increasing distances from the reference plane: in fact, the area colored in blue (that corresponds to a standard deviation no greater than 0.002 m) on the image frame progressively shrinks. At the same time, the area with a standard deviation lower than 0.005 m gets smaller and smaller, implying that at a distance of 1.5 m the frame cannot be used entirely. Nevertheless, as the distance from the reference plane increases other objects are present in the scene (e.g., the plug at 3.7 m that corresponds to the red blot visible in Figure 12).

## 4. Test with Fusion Libraries 

The Kinect Fusion Libraries were released by Microsoft on March 2013 and inserted in the SDK for Kinect 2.0 on September 2014. They allow one to quickly create a 3D mesh model of an object or a small scene by simply holding the sensor and moving it around. These libraries create a single shaded 3D model, by integrating and merging depth data, while tracking the sensor pose [19,37]. Although the depth data delivered by Kinect 2.0 is more complete that the ones delivered by the previous version, the model created using a single depth frame could be very incomplete. The documentation about the new release of the Fusion Libraries is not as complete as the one distributed for the Kinect 1.0, however the implementation is quite similar. Firstly, depth data are converted into point clouds. Each point clouds is projected into a 2D space and the normals are computed. Then an Iterative Closest Points (ICP) [38,39], algorithm implemented on a Graphic Processing Unit (GPU) is used to align the point clouds. This is possible because the scene is acquired by different viewpoints, as the sensor pose (location and/or orientation) changes instantaneously. The new Fusion Libraries are more robust than Fusion 1.0 because the camera pose estimation (which is a crucial step, necessary for the following volume reconstruction) can be performed by aligning two overlapping point clouds or recovered using directly the depth data and aligned them to the previously reconstructed volume. When the new depth frame is aligned to the previously acquired data, the new depth measurements are added to the 3D volume; if the new observations are recognized to lie on the object surface (applying a Truncated Signed Distance Function), they are merged to the model, averaging data that corresponds to the same cells in the volume space. When the processing is complete, another frame can be processed.

**Figure 13:**
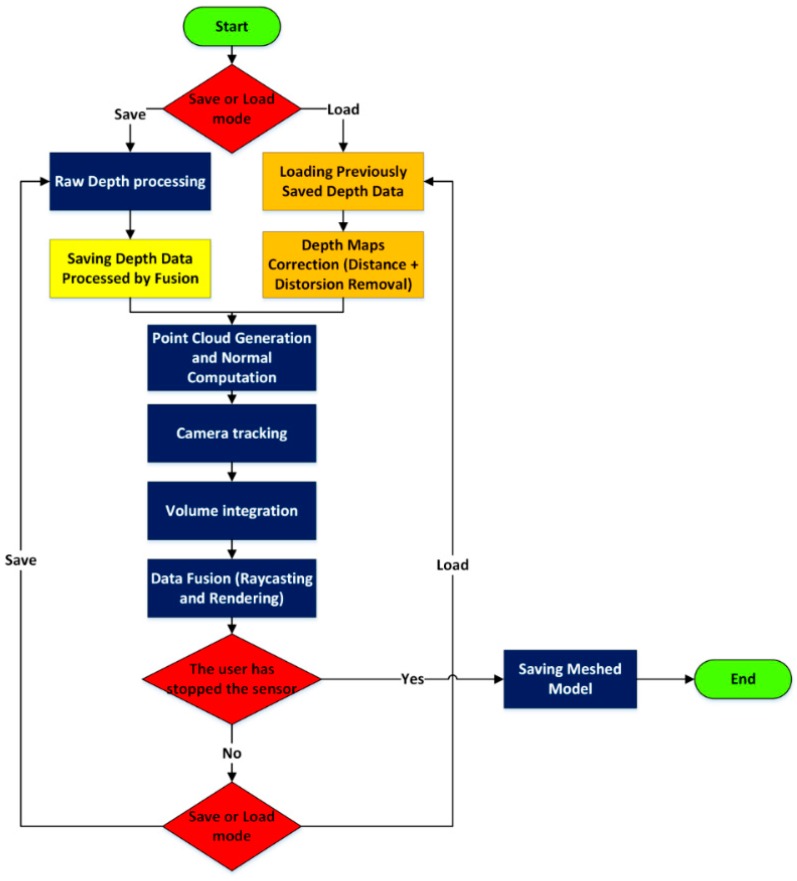
Modified Fusion pipeline for the depth data correction.

Within the Fusion Libraries only the generic values for the depth camera focal length and for the principal point are taken into account. It is important also to correct the images from lens distortions. Therefore, the Fusion Libraries pipeline has been modified in order to correct each frame before the model reconstruction. The coded software is characterized by two main phases (see Figure 13): during the first step the depth frame processed by Fusion Libraries are saved. Then the data are load and each frame is corrected from lens distortions, applying the coefficients of the Brown model estimated during the calibration phase (see Table 3). 

A test was designed, to understand the impact of the proposed correction. A small statue (0.20 m height and 0.15 m wide) has been surveyed by moving the Kinect around it. The reference model has been created photogrammetrically, acquiring 99 images around the statue and processing them with the commercial software package Agisoft Photoscan (version 1.1.4) [40]. The images were acquired with a Canon EOS1100D camera with 35 mm fixed focal length, using a 4272 × 2848 pixel resolution. The meshes created with the Kinect and the reference 3D model have been globally registered using the ICP algorithm.

**Figure 14:**
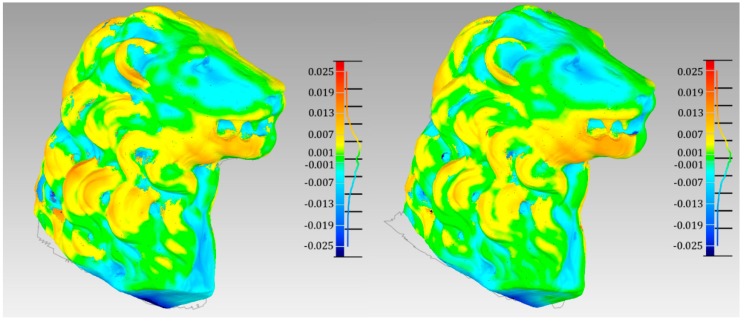
3D comparison (shortest differences) between the reference model and the Kinect Fusion mesh (**left**) and the Fusion model with the IO parameters correction (**right**).

The Euclidean distances between the reference model and the ones created using the Kinect 2.0 are shown in Figure 14. Is quite evident that the model created by the Kinect is very smooth, in fact the differences are higher (even few centimeters), in correspondence of the lion’s mane. High differences can be noticed also in correspondence of the jaws, underling a problem due to the ICP alignment between the Kinect 2.0 models and the photogrammetric one. Nevertheless, some slight improvements are obtained using distortion free depth frames; in fact, the green area enlarges, which corresponds to differences lower than 0.001 m. 

Globally, the improvement is quite small, but it is important to notice that the model was located in the center of the frame, in order to maintain the minimum distance necessary for the depth acquisition.

Because of the limited dimension of the surveyed object and the fact that it was centered with respect to the depth sensor, the effect due to the distortion correction is small. However, the achieved results are promising and show how it is important to correct the depth data before the 3D modelling creation, especially if the whole frame is used (*i.e*., small scene like an office corner).

## 5. Conclusions

The Kinect has shown since its release on the market great potential for research use because it allows combining visual and depth data, attracting interest from a wide variety of fields. It can be remotely controlled by a PC and used as a measurement system, by delivering a large amount of data at a high frame rate. However, it is a low-cost device (initially designed as a game controller), so it is fundamental to investigate its precision and accuracy. To this end a straightforward calibration procedure has been performed. Firstly, the stability of the imaging sensors (RGB and IR) was evaluated. In both cases the superior performances of the Kinect 2.0 was quite evident. In order to use the Kinect as a low-cost measurement systems it is important to evaluate the depth error. A calibration procedure has been realized, defining sensor error as a function of the distance between the device and the object. It can be used to correct the whole image (pixel by pixel), in dependence of the measured depth. Both the error produced by Kinect 1.0 and its noise can be described as a second order polynomial functions. Kinect 2.0 is characterized by an error and a precision that increase linearly. All the experimental results have been statistically tested and the error model functions estimated. Second order effects that characterized the Kinect 2.0 depth frames have been investigated too.

Distortion correction has been applied to each depth frame used by the Fusion libraries, underlying how it is possible to obtain more correct 3D models by adding a new function within the code released by Microsoft. The object used was quite small and located in the center of the sensor plane; however, the obtained results are quite promising and showed the importance of such correction if the entire depth frame is used.
